# Distribution of GABAergic cells in the inferior colliculus that project to the thalamus

**DOI:** 10.3389/fnana.2014.00017

**Published:** 2014-03-31

**Authors:** Jeffrey G. Mellott, Nichole L. Foster, Kyle T. Nakamoto, Susan D. Motts, Brett R. Schofield

**Affiliations:** ^1^Department of Anatomy and Neurobiology, Northeast Ohio Medical UniversityRootstown, OH, USA; ^2^School of Biomedical Sciences, Kent State UniversityKent, OH, USA; ^3^Department of Physical Therapy, Arkansas State UniversityJonesboro, AR, USA

**Keywords:** tectothalamic, medial geniculate, inhibition, GAD, auditory system

## Abstract

A GABAergic component has been identified in the projection from the inferior colliculus (IC) to the medial geniculate body (MG) in cats and rats. We sought to determine if this GABAergic pathway exists in guinea pig, a species widely used in auditory research. The guinea pig IC contains GABAergic cells, but their relative abundance in the IC and their relative contributions to tectothalamic projections are unknown. We identified GABAergic cells with immunochemistry for glutamic acid decarboxylase (GAD) and determined that ~21% of IC neurons are GABAergic. We then combined retrograde tracing with GAD immunohistochemistry to identify the GABAergic tectothalamic projection. Large injections of Fast Blue, red fluorescent beads or FluoroGold were deposited to include all subdivisions of the MG. The results demonstrate a GABAergic pathway from each IC subdivision to the ipsilateral MG. GABAergic cells constitute ~22% of this ipsilateral pathway. In addition, each subdivision of the IC had a GABAergic projection to the contralateral MG. Measured by number of tectothalamic cells, the contralateral projection is about 10% of the size of the ipsilateral projection. GABAergic cells constitute about 20% of the contralateral projection. In summary, the results demonstrate a tectothalamic projection in guinea pigs that originates in part from GABAergic cells that project ipsilaterally or contralaterally to the MG. The results show similarities to both rats and cats, and carry implications for the role of GABAergic tectothalamic projections vis-à-vis the presence (in cats) or near absence (in rats and guinea pigs) of GABAergic interneurons in the MG.

## Introduction

The inferior colliculus (IC) is a large midbrain structure that is a target of both ascending and descending auditory pathways as well as a source of projections to numerous areas. Like most brain areas, the IC contains a mixture of excitatory and inhibitory neurons. In the IC, the two main classes of neurons are glutamatergic and GABAergic, and most or perhaps all IC neurons belong to one of these two classes (Ito and Oliver, 2012). Unlike many other brain areas, the GABAergic cells provide a significant contribution to the output pathways from the IC (Appell and Behan, 1990; Wenstrup, 2005). The largest and best studied pathway is the tectothalamic projection from the IC to the medial geniculate nucleus (MG), the auditory center of the thalamus. Quantitative analyses have been completed in two species, where the GABAergic cells constitute about 40% of the tectothalamic pathway in rats (Peruzzi et al., 1997) but only 20% of the pathway in cats. The difference between the two species is particularly interesting given that GABAergic cells reportedly constitute about 20–25% of IC cells in both species (Oliver et al., 1994; Merchán et al., 2005). In other words, the GABAergic cells in the tectothalamic pathway appear to reflect the overall proportion of GABAergic cells in the IC in cats, but in rats the GABAergic cells are disproportionately prominent in the tectothalamic pathway.

The variability in GABAergic composition of the tectothalamic pathway makes it difficult to generalize the results to other species. Guinea pigs are widely used in auditory research and have a prominent population of GABAergic cells in the IC (Foster and Schofield, 2014). Preliminary studies have demonstrated that some of these GABAergic cells project to the MG (Mellott et al., 2011). However, quantitative analyses have not been done, so both the percentage of IC cells that are GABAergic, and the percentage of GABAergic cells in the tectothalamic projection, remain unknown. For the present study, we used immunochemistry and retrograde tracers to examine the GABAergic cells in guinea pig IC and their contributions to IC-MG projections. The results suggest that GABAergic cells make up about 21% of IC neurons, similar to the reports in rats and cats. Furthermore, GABAergic cells appear to constitute ~22% of the IC cells that project to the MG. This value is closer to that reported in cats (~20%; Winer et al., 1996) than in rats (~40%; Peruzzi et al., 1997), and has implications for the relative contributions of ascending vs. intrathalamic sources of GABA for integration of inhibitory signals in the MG.

Coomes et al. (2002) suggested that rats may have a higher percentage of GABAergic cells in the tectothalamic pathway to compensate for the near absence of MG interneurons in that species (<1% of MG neurons; Winer and Larue, 1988). In contrast, cats have more interneurons (about 25% of MG neurons; Huang et al., 1999) and relatively fewer GABAergic tectothalamic cells. If this relationship is true across species, one would predict that guinea pigs, which have few MG interneurons, would have many GABAergic tectothalamic cells (i.e., guinea pigs should be like rats).

We addressed several questions regarding GABAergic IC cells in guinea pigs: What percentage of IC neurons are GABAergic? What proportion of the tectothalamic cells are GABAergic? We asked additional questions that have been addressed little or not at all in past studies. Does the projection from the IC to the contralateral MG have a GABAergic component? If so, what proportion of these cells are GABAergic?

## Materials and methods

All procedures were conducted in accordance with the Institutional Animal Care and Use Committee and NIH guidelines. Results are described from nine adult pigmented guinea pigs (Elm Hill Labs; Chelmsford, MA, USA) of either sex weighing 389–867 g. Efforts were made to minimize the number of animals and their suffering.

### Surgery

Each animal was anesthetized with isoflurane (4–5% for induction, 1.75–3% for maintenance) in oxygen. The head was shaved and disinfected with Betadine (Purdue Products L.P., Stamford, CT, USA). Atropine sulfate (0.08 mg/kg i.m.) was given to minimize respiratory secretions and a one time injection of Ketofen (ketoprofen, 3 mg/kg i.m.; Henry Schein, Melville, NY 11747, USA) was given for post-operative pain management. Moisture Eyes PM ophthalmic ointment (Bausch & Lomb, Rochester, NY, USA) was applied to each eye to protect the cornea. The animal's head was positioned in a stereotaxic frame. Body temperature was maintained with a feedback-controlled heating pad. Sterile instruments and aseptic techniques were used for all surgical procedures. An incision was made in the scalp and the surrounding skin was injected with Marcaine (0.25% bupivacaine with epinephrine 1:200,000; Hospira, Inc., Lake Forest, IL, USA), a long-lasting local anesthetic. A craniotomy was made over the desired target coordinates using a dental drill. Following the tracer injection, Gelfoam (Harvard Apparatus, Holliston, MA, USA) was placed in the craniotomy site and the scalp was sutured. The animal was then removed from the stereotaxic frame and placed in a clean cage. The animal was monitored until it could walk, eat and drink without difficulty.

### Retrograde tracers

Three fluorescent tracers were used: (1) red fluorescent RetroBeads (“red beads”), injected without dilution; (Luma-Fluor, Inc., Naples, FL, USA); (2) Fast Blue, 5% in water (EMS-Chemi GmbH, Gross Umstadt, Germany); (3) FluoroGold, 4% in water (FluoroChrome, Inc., Englewood, CO, USA). Tracers were deposited into the medial geniculate body (MG) via stereotaxic coordinates. A Hamilton microsyringe (1 μl; Hamilton, Reno, NV, USA) with a sterile needle was used to deposit one of the tracers into the MG. Each syringe was dedicated to a single tracer. In order to include as much of the MG as possible while limiting the spread of tracer into neighboring nuclei, the number of deposits and the volume at each site were designed to account for the diffusibility of each tracer (Schofield, 2008). The RB tracer diffuses very little and so was deposited at 2–4 locations in the MG whereas FB and FG, which diffuse more readily, were each deposited at 1 central location (Table 1).

**Table 1:** **A list of the tracers injected in the left (L) and right (R) MG in each case along with injection parameters and extent of injection sites**.

**Case**	**Side**	**Tracer**	**# of deposit sites**	**Total volume**	**Extent of injection site**				
					**MGv**	**MGd**	**MGsg**	**MGm**	**other**
GP632	L	RB	1	0.2μl	x	x	x	x	0
GP632	R	FG	1	0.15μl	x	x	x	x	SC
GP633	L	RB	2	0.4μl	x	x	x	x	0
GP633	R	FG	1	0.08μl	x	x	x	x	APT
GP636	L	RB	2	0.4μl	x	x	x	x	0
GP636	R	FG	1	0.05μl	x	x	x	x	0
GP638	L	RB	2	0.5μl	x	x	0	x	0
GP638	R	FB	1	0.08μl	x	x	(x)	0	LG, LP
GP640	L	RB	4	0.4μl	x	x	x	x	0
GP640	R	FB	1	0.08μl	x	x	x	x	0

*FB, Fast Blue; FG, FluoroGold; RB, red RetroBeads. Total Volume: total volume of tracer injected. Extent of injection site: x indicates tracer filled a substantial portion of the region indicated; (x) indicates minor involvement; 0 indicates no spread of tracer into the area. Abbreviations: APT, anterior pretectum; LG, lateral geniculate nucleus; LP, lateral posterior nucleus; SC, superior colliculus*.

### Perfusion and tissue processing

Animals were checked daily after surgery and monitored for health. Five days after surgery, the animal was deeply anesthetized with isoflurane and perfused transcardially with Tyrode's solution, followed by 250 ml of 4% paraformaldehyde in 0.1M phosphate buffer, pH 7.4 and then by 250 ml of the same fixative with 10% sucrose. The brain was removed and stored at 4°C in fixative with 25–30% sucrose for cryoprotection. The following day the brain was prepared for processing by removing the cerebellum and blocking the remaining piece with transverse cuts posterior to the superior olive and anterior to the auditory cortex. Each piece of tissue was frozen and cut on a sliding microtome into 40 or 50 μm thick transverse or sagittal sections that were collected serially in six sets.

Putative GABAergic cells were stained with immunochemistry for glutamic acid decarboxylase (GAD) (Nakamoto et al., 2013). We tested several antibodies for efficacy in guinea pig IC tissue. GABAergic neurons typically contain two forms of GAD—GAD65 and GAD67—that differ in molecular weight and in cellular distribution (as well as other properties). GAD67 is typically found throughout the cell whereas GAD65 is usually concentrated in terminals. Antibodies to GAD67 have been used routinely for identifying GABAergic cells in the auditory brainstem (e.g., Ito et al., 2011; Ito and Oliver, 2012; Stange et al., 2013). In preliminary studies, we stained guinea pig IC tissue with three different antibodies. Two of the antibodies are selective for GAD67: (1) Santa Cruz GAD-67 (H-101): sc-5602; (2) Millipore AntiGAD67, clone 1G10.2 (Cat. # MAB5406). The third antibody recognizes both GAD65 and GAD67 (Chemicon anti-GAD AB5992). Visual inspection of stained IC cells indicated that somatic staining was similar or possibly slightly more robust with the Millipore anti-GAD67 than with the other 2 antibodies. Some IC neurons have GABAergic boutons on their somas (Merchán et al., 2005); intense staining of these boutons makes identification of somatic staining somewhat more difficult. All 3 antibodies that we tested stained boutons in the IC; the Millipore antibody again proved advantageous for the present study because it stained the boutons somewhat less intensely than the other 2 antibodies, so assessing the somatic label was relatively simpler. The results presented in this paper are based on staining with the Millipore anti-GAD67 antibody. Briefly, the sections were pretreated with normal goat serum to limit non-specific labeling and 0.1% Triton X-100 to improve penetration of reagents in to the tissue, then exposed (1–2 days at 4°C) to mouse anti-GAD polyclonal antibody (GAD67; #MAB5406 Millipore, diluted 1:1000 to 1:100). Then the sections were treated with 1% biotinylated goat anti-mouse antibody (Vector Laboratories, Burlingame, CA, USA: BA-9200) and labeled with streptavidin conjugated to the fluorescent marker (AlexaFluor 488 [AF488, green] Invitrogen, Carlsbad, CA, USA). In order to assess the percentage of IC neurons that are GABAergic, we double stained a series of sections from four animals with anti-GAD (as above) and a neuronal nucleus-specific antibody (anti-Neu-N; #ABN78 Millipore, diluted to 1:500). Neu-N was visualized with a secondary antibody conjugated to AlexaFluor 750 (AF750; Invitrogen, Carlsbad, CA, USA). In all animals, one series of sections was stained with antibodies to brain nitric oxide synthase (bNOS) to identify IC subdivisions (Coote and Rees, 2008). Stained sections were mounted on gelatin-coated slides, allowed to dry and coverslipped with DPX (Sigma).

### Data analysis

#### Cytoarchitecture

Subdivisions of the MG were identified by their patterns of staining with cytochrome oxidase (Anderson et al., 2007). IC subdivisions were identified by the differential expression of brain nitric oxide synthase (bNOS), as detailed in Coote and Rees (2008). The borders of the ICc were clarified by observation at high power to identify disc-shaped cells that stain for bNOS and that are characteristic of the ICc (Coote and Rees, 2008).

#### Immunochemistry

Immunostaining revealed GAD-immunoreactive (GAD+) cells and boutons throughout the IC. Immunopositive cells were labeled intensely and were readily distinguished from immunonegative cells. The GAD immunostain was also readily visible in tracer-labeled cells, making it straightforward to distinguish GAD+ vs. GAD-negative staining in the retrogradely-labeled cells, including cells that contained two different retrograde tracers.

The location and extent of each injection site was determined by comparison of the tracer deposit with borders of MG subdivisions identified in sections stained for cytochrome oxidase (Anderson et al., 2007). Results from eight injections (4 RB; 2 FB; 2 FG) that also had robust immunostaining were used for quantitative analysis (we excluded GP632 because the FG injection spread caudally into the superior colliculus, which receives projections from the IC). Labeled cells in the IC were plotted with a Neurolucida reconstruction system (MBF Bioscience, Williston, VT, USA) attached to a Zeiss Axioplan II microscope (Carl Zeiss MicroImaging, Inc., Thornwood, NY, USA). For each case, every labeled cell was plotted in both left and right IC in two transverse sections. One section was selected that went through the “center” of the IC (along the rostro-caudal axis); such a section is commonly used to summarize connections of the IC subdivisions because it contains substantial portions of central, dorsal and lateral IC subdivisions. Each combination of tracer and immunolabel was plotted with a unique marker. The results of these plots were used for a quantitative summary of the distributions of the labeled cells.

In some cases, the anti-GAD staining did not fully penetrate the tissue, resulting in a central layer in the section where GAD staining was absent. Data from these cases were plotted with the Neurolucida system and a 63X objective (NA = 1.4), with special attention to focusing on the center of the soma when plotting the symbol for a particular cell. This approach provides sufficient resolution in the z plane (section depth) to allow meaningful filtering of the data by depth. After the data were plotted, the X, Y, and Z coordinates of all markers from each subdivision of each tissue section were exported from Neurolucida to Microsoft Excel and sorted based on the Z coordinate. The depth of penetration of the GAD labeling was assessed under the 63X objective for each subdivision of each section to determine the range of depths (measured from the top surface of the section) where GAD staining was robust. This yielded two zones of data from each section (1 associated with each surface), and a central zone that was not stained with GAD. All markers in the central, unstained zone were excluded from further analyses.

Neu-N immunopositive cells were quantified in four cases. For each case, three sections were chosen to include substantial portions of the central nucleus, lateral cortex, and dorsal cortex. Two cases were cut in the transverse plane and two cases were cut in the parasagittal plane, so cells from the medial, lateral, rostral, and caudal extremes of the IC were included in the sample. To control for incomplete penetration of the immunostains, cell counts were limited to the tissue within 5 μm of each surface of the section (as described above for counts of GAD staining).

Figures showing the distribution of labeled cells were created with Neurolucida software (MBF Bioscience) and refined with Adobe Illustrator (Adobe Systems, Inc., San Jose, CA, USA). Photomicrographs were captured with one of three fluorescence microscopes: (1) a Zeiss AxioImager Z1 fluorescence microscope and AxioCam HRm or HRc cameras (Zeiss); (2) a Zeiss Axioskop fluorescence microscope and Magnafire camera (Optronics, Goleta, CA, USA); (3) a Zeiss AxioImager Z2 equipped with a Microfire camera (Optronics). Both AxioImager microscopes were equipped with an Apotome (Zeiss) structured illumination system that allows for optical sectioning in the z plane (similar to confocal imaging). Imaging with the Apotome was used to clarify the presence of specific fluorescent labels in any instances of ambiguity. Adobe Photoshop (Adobe Systems) was used to add scale bars, crop images, erase background around tissue sections, adjust intensity levels and colorize monochrome images.

## Results

We used immunolabeling for GAD to identify GABAergic IC cells and then combined retrograde tracing with the immunostain to identify GABAergic IC cells that project to the MG. We first describe the number of GABAergic cells in the IC (as a percentage of IC Neu-N-immunopositive neurons). We then describe the injection sites, the distribution and number of GAD-immunopositive (“GAD+”) and GAD-immunonegative (GAD-negative) cells that were labeled by retrograde transport from the MG.

### GAD immunolabeling

**Figure 1:**
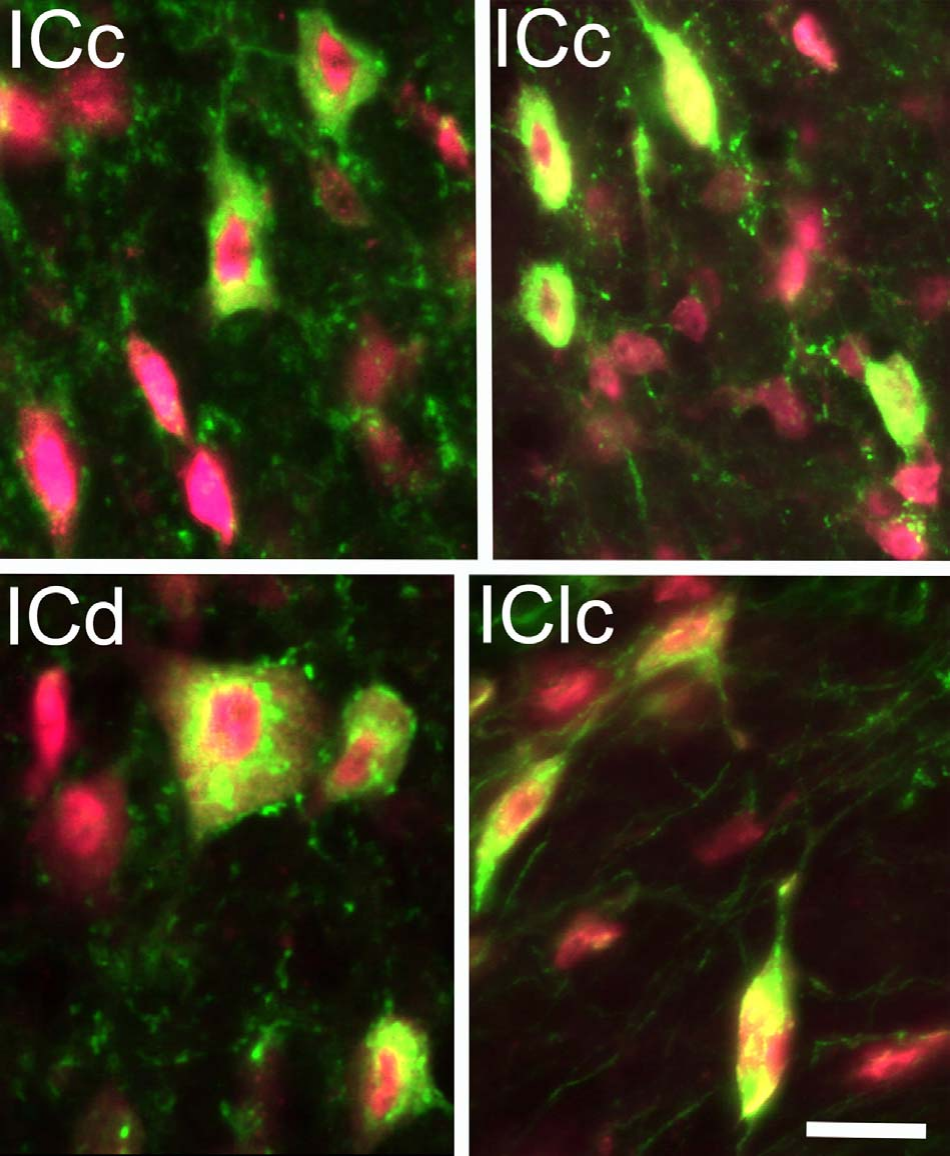
**Photomicrographs showing GAD+ cells (green) in the inferior colliculus (IC)**. Neuronal nuclei, and to a lesser extent the cytoplasm, were stained with anti-NeuN (magenta). The fluorescent image from each channel is merged in this plate; viewing the channels separately confirmed that all the GAD+ cells were also immunopositive for NeuN. GAD+ cells were found throughout the subdivisions of the IC and exhibited a wide range of sizes and shapes. Examples are shown from the IC central nucleus (ICc); IC dorsal cortex; (ICd) and IC lateral cortex (IClc). Scale bar = 20 μm.

GAD+ cells were present in each subdivision of the IC. Nearly all GAD+ cells (14,222/14,257 = 99.8%) were also stained for NeuN, confirming their identity as neurons (Figure 1). We interpret these immunopositive cells as GABAergic cells. We noted a wide range of sizes in all IC subdivisions (~8 μm to ~40 μm in major diameter), suggesting broad similarity with GABAergic IC cells described in other species. On the whole, 21% of the IC cells immunopositive for Neu-N were also immunopositive for GAD. Thus, 21% of IC neurons in guinea pigs appear to be GABAergic.

### Injection sites for retrograde transport

**Figure 2:**
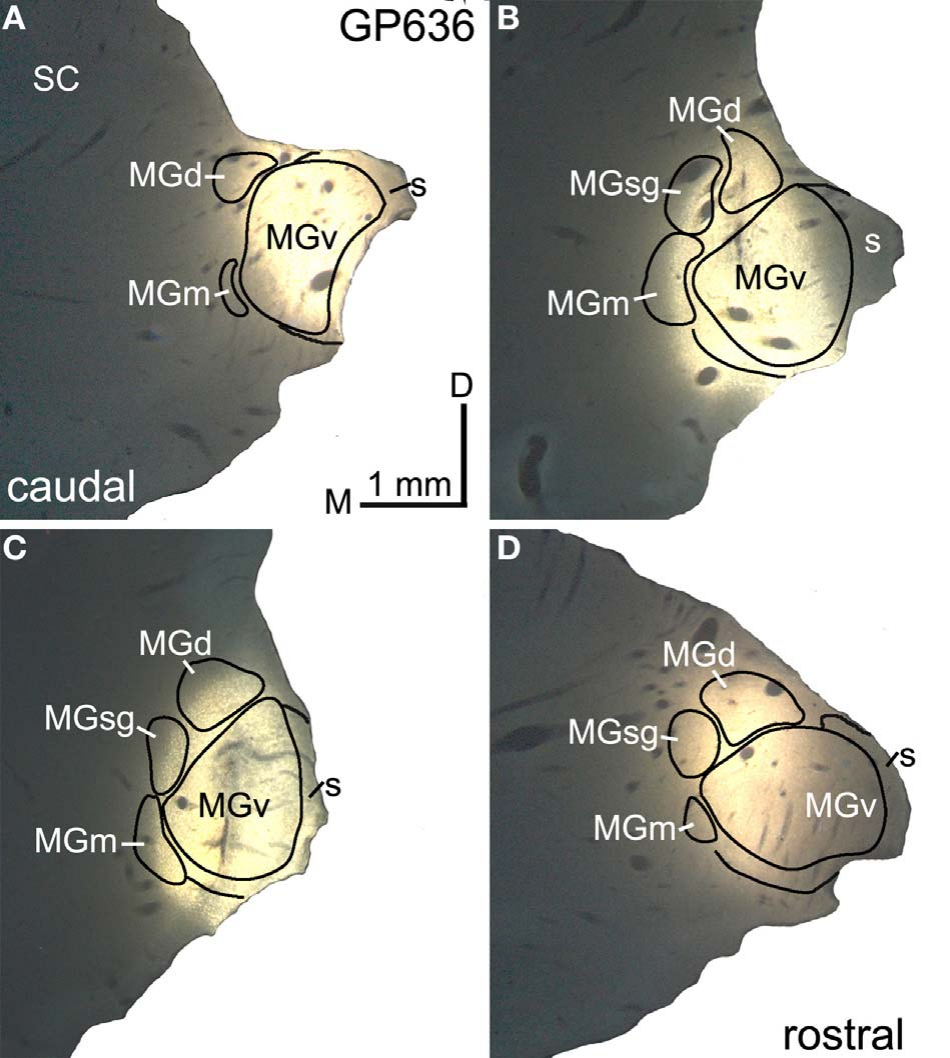
**Photomicrographs showing a representative deposit of FluoroGold in the medial geniculate body (MG). (A–D)** A series of transverse sections through the right MG, arranged from caudal to rostral. The injection site involves all of the major subdivisions of the MG. GP636. D, dorsal; MGd, dorsal division of the MG; M, medial; MGm, medial division of the MG; s, shell of the MG; SC, superior colliculus; MGsg, suprageniculate division of the MG; MGv, ventral division of the MG.

Figure 2 shows a representative injection of FluoroGold (FG) in the right MG of GP636. The injection site includes large portions of all MG subdivisions (Figures 2A–D). Table 1 summarizes the extent of the injection sites in each case. In case GP638 R the tracer spread into the dorsal lateral geniculate nucleus and lateral posterior nucleus and in case GP 633R it spread into the anterior pretectal nucleus. There is no evidence that the IC projects to these nuclei, so we interpret the labeled IC cells as projecting to the MG. In GP632 R, the FG injection spread caudally to invade the right superior colliculus (SC). The spread was very limited, but FG-labeled cells were present in the contralateral (left) SC, suggesting that uptake occurred in the right SC (the labeled cells in the left SC being cells that project from one SC to the other via the commissure of the SC). The IC also projects to the SC, and this projection includes GABAergic cells in the IClc (Appell and Behan, 1990; Mellott and Schofield, 2012). The number of such cells is very small in the IClc (and even smaller in the ICc and ICd), so the majority of labeled cells in the IC in the case GP632 almost certainly project to the MG. Nonetheless, we excluded case GP632 from our quantitative analyses.

Aside from the differences discussed above, the results were similar qualitatively across cases and across tracers. Each injection labeled a large number of cells in the IC as well as fewer cells in other areas reported to project to the MG (e.g., cochlear nuclei: Anderson et al., 2006; superior olivary complex and sagulum: Aitkin et al., 1981). Within the IC, cells labeled by a given injection were distributed bilaterally, with the majority (average = 90%; *n* = 9938 cells) located ipsilateral to the injection site. On both sides, retrogradely labeled cells were located in all IC subdivisions, consistent with injection sites that involved all MG subdivisions (Anderson et al., 2007).

There were quantitative differences between experiments in the number of labeled cells in the IC. Not surprisingly, a given tracer labeled more cells when the injection site was larger. This is demonstrated most clearly by RB injections. The RB injection in GP632 spread into all 4 MG subdivisions, but nonetheless excluded parts of each subdivision. In subsequent cases, we injected 2–2.5 times the volume of red beads, resulting in a much greater number of RB-labeled cells. A second distinction related to differences between the tracers. The RB tracer labeled the most IC cells even though the injection sites appeared more restricted than those obtained with FB or FG. This is a typical result with these tracers and results in part from their different diffusion properties (Schofield et al., 2007; Schofield, 2008).

### Morphology and distribution of IC cells that project to the ipsilateral or contralateral MG

**Figure 3:**
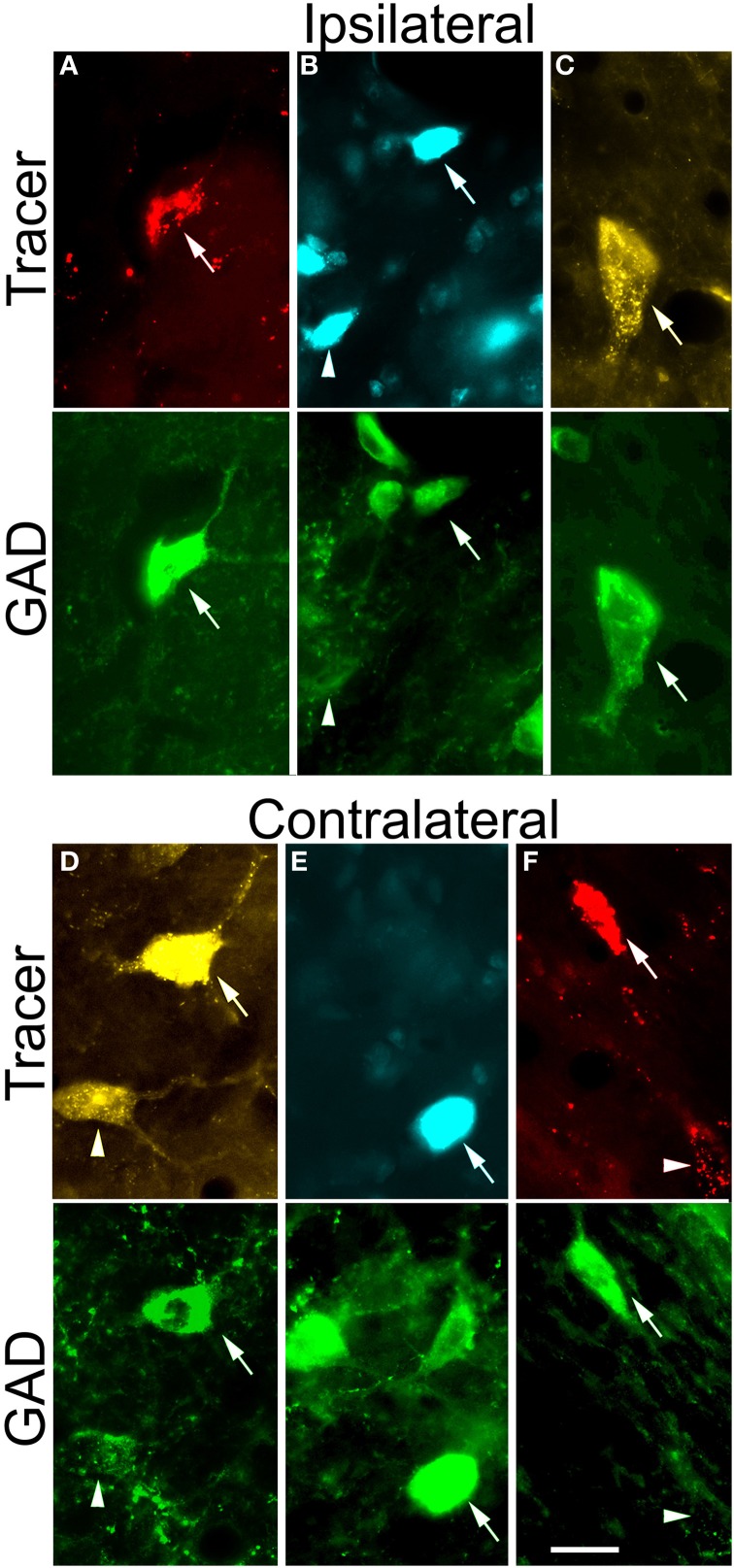
**Paired photomicrographs showing retrogradely-labeled tectothalamic cells that are GAD-immunopositive (GAD+) or GAD-negative and that project to the ipsilateral MG (A–C) or to the contralateral MG **(D–F)****. The top row in each pair shows cells retrogradely labeled by red beads (RB), FluoroGold (FG), or Fast Blue (FB). The bottom row in each pair shows the same cells viewed for immunoreactivity to GAD, visualized with AlexaFluor 488 (green). In all panels, arrows indicate cells that were double-labeled with a tracer and the GAD immunostain. Arrowheads indicate cells that were retrogradely labeled but immunonegative for GAD. **(A–C)** Cells labeled by retrograde transport from the *ipsilateral* MG. Examples are shown from each IC subdivision. **(A)** ICc: RB-labeled cell (GP638). **(B)** ICd: FB-labeled cells (GP638). **(C)** IClc: FG-labeled cell (GP636). **(D–F)** Cells labeled by retrograde transport from the *contralateral* MG. Examples are shown from each IC subdivision. **(D)** ICc: FG-labeled cells (GP636), **(E)** ICd: FB-labeled cell (GP640). **(F)** IClc: RB-labeled cell (GP636). Scale bars = 20 μm. ICc, IC central nucleus; ICd, IC dorsal cortex; IClc, IC lateral cortex.

**Figure 4:**
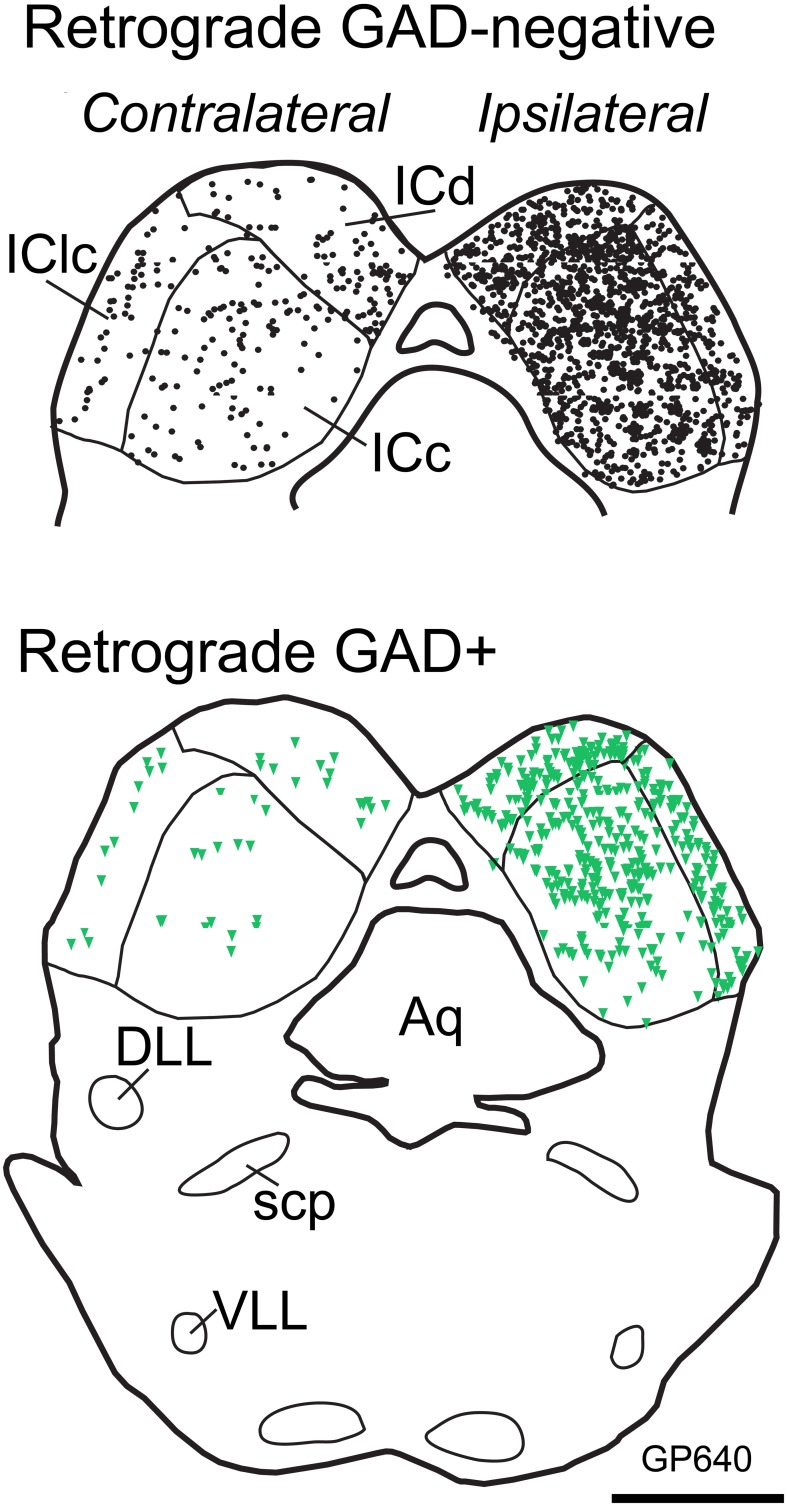
**Plots of transverse sections illustrating the distribution of GAD-negative (Top; black circles) and GAD+ (Bottom; green triangles) IC cells that were labeled by an injection of Fast Blue into the right MG**. Each symbol represents one retrogradely-labeled cell. Dorsal is up. Scale bar = 1 mm. Aq, aqueduct; DLL, dorsal nucleus of the lateral lemniscus; IC, inferior colliculus; ICd, dorsal cortex of the IC; IClc, lateral cortex of the IC; scp, superior cerebellar peduncle; VLL, ventral nucleus of the lateral lemniscus. Case GP640.

Following a typical large injection, we observed thousands of tracer-labeled cells throughout the IC. The tracer-labeled cells were morphologically heterogeneous (Figure 3). While many could not be assigned to a morphologic class with certainty, the population included both disc and stellate cells in the ICc as well as stellate cells of a wide range of sizes in the other subdivisions. GAD immunofluorescence was readily identified in a subset of the cells that contained a retrograde tracer (Figure 3; arrows). These cells were interpreted as GABAergic cells that project to the MG. GAD-negative retrogradely-labeled cells were often in close proximity to GAD+ cells (Figure 3; arrowheads), suggesting that the immunonegative cells are non-GABAergic and not immunonegative as a result of inadequate GAD staining. While we did not analyze morphology in detail, it appeared that a similar range of morphologies was present in both the GAD+ and the GAD-negative populations. Figure 4 shows the distribution of tracer-labeled cells in the IC after a deposit of Fast Blue in the right MG. The GAD-negative and GAD+ populations were intermingled, but are shown in separate plots for clarity (Figure 4A: GAD-negative tracer-labeled cells; Figure 4B: GAD+ tracer-labeled cells). The majority of the tracer labeled cells in the IC were located ipsilateral to the injection (average = 90%; *n* = 9938 cells in 8 experiments). A similar ipsilateral dominance characterized both the GAD-negative population (average = 89%, *n* = 7713 cells in 8 experiments) and the GAD+ population (average = 93%, *n* = 2225 cells in 8 experiments).

**Figure 5:**
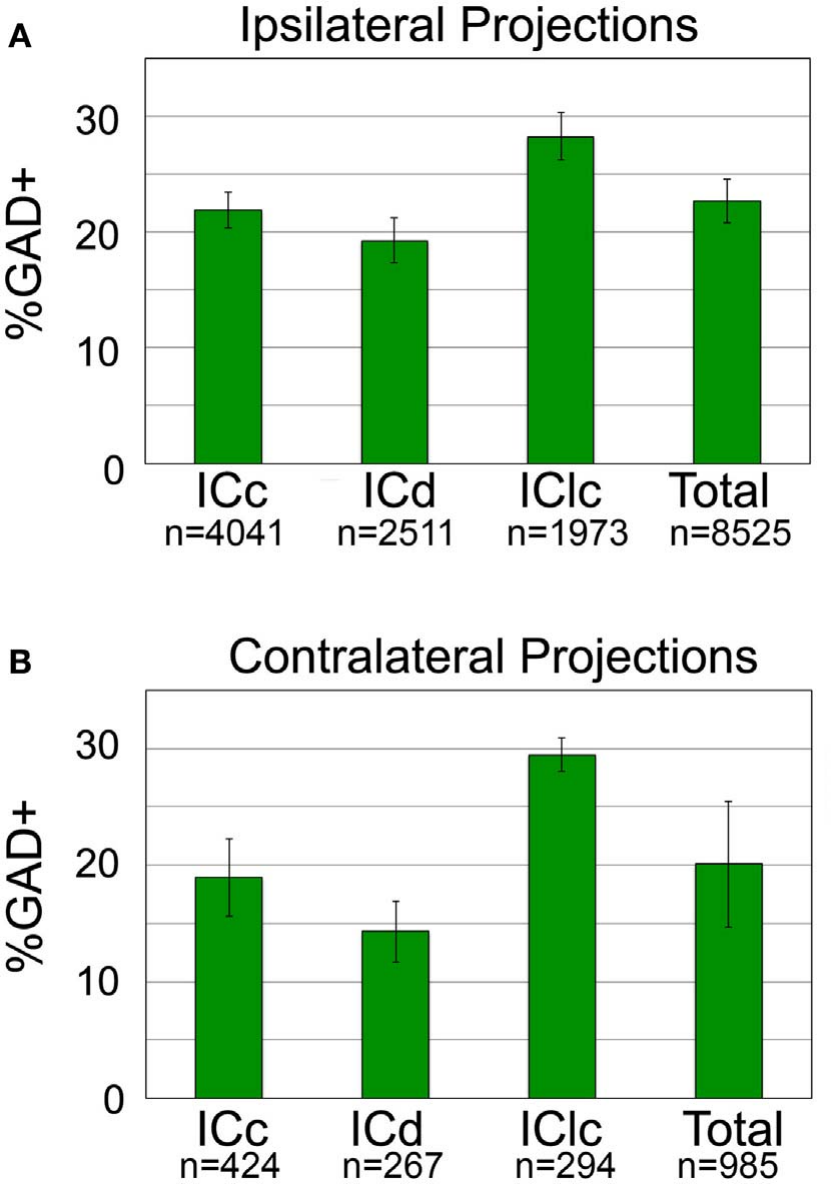
**Percentage of tectothalamic cells that are GAD+ in each subdivision of the IC and for the IC overall. (A)** Percentages for the ipsilateral projection. **(B)** Percentages for the contralateral projection. “*n*” indicates the number of tracer-labeled cells in each category. Error bars represent the SEM. ICc, IC central nucleus; ICd, IC dorsal cortex; IClc, IC lateral cortex.

We calculated the proportion of tracer-labeled cells in each IC subdivision that was GAD+. Figure 5A shows the results for the ipsilateral pathway. Overall, GAD+ cells constitute about 22% of the ipsilateral tectothalamic pathway. The GAD+ proportion varied somewhat by IC subdivision, being highest for the IClc (28%) and lowest for the ICd (19%) (Figure 5A). Analysis of the contralateral pathway showed similar results (Figure 5B). Overall, GAD+ cells constitute 20% of the contralateral tectothalamic pathway. Variations among the IC subdivisions paralleled those of the ipsilateral pathway: the highest GAD+ percentage occurred in the IClc (29%) and the lowest GAD+ percentage occurred in the ICd (14%).

## Discussion

The current study identifies GABAergic projections from the IC to the ipsilateral and contralateral MG in guinea pigs. Overall, 22% of the tectothalamic cells are GABAergic. GABAergic tectothalamic cells occur in each subdivision of the IC, constituting 14–29% of the projection, depending on the subdivision. Thus, guinea pigs, like cats and rats, have a robust GABAergic projection from each IC subdivision to the MG. These widespread origins suggest that the GABAergic projections contribute to the full range of auditory functions associated with the tectothalamic pathway. In addition to the ipsilateral projection, there is a smaller contralateral tectothalamic projection. GABAergic cells make up 20% of this crossed projection.

### Technical considerations

We identified IC subdivisions according to established criteria in guinea pigs (Coote and Rees, 2008). As noted by the latter authors, some borders, particularly between ICc and ICd, are difficult to distinguish with bNOS. It may be that these subdivisions do not exhibit a sharp border, a possibility suggested with other species and techniques (Faye-Lund and Osen, 1985; Herbert et al., 1991; Gonzalez-Lima and Jones, 1994; Malmierca et al., 1995; Coote and Rees, 2008; Song et al., 2011). In the present study, altering the location of the borders would change the relative contributions of different IC subdivisions to the tectothalamic projections, but it would not change the finding that all subdivisions have GABAergic tectothalamic cells nor would it substantially alter the calculations of the GABAergic contributions to these projections.

The antibody we used for GAD staining has been validated in guinea pigs (Xiong et al., 2008; Nakamoto et al., 2013). Controls included western blot analysis as well as primary and secondary omissions. In all cases, staining was robust and matched previous descriptions of GABAergic cells in the IC as well as other brainstem nuclei (e.g., Adams and Mugnaini, 1984; Kulesza and Berrebi, 2000; Ito et al., 2009). We saw no evidence of false positive staining, even at the highest concentration of primary antibody. False negative staining is harder to rule out. Some previous studies of IC GABAergic cells (e.g., Oliver et al., 1994) used an antibody to GAD67 as in the present study, whereas others (e.g., Winer et al., 1996; Peruzzi et al., 1997) used an antibody to GABA (and different fixative, as needed for anti-GABA staining). Importantly, Oliver et al. (1994) used both anti-GAD67 and anti-GABA antibodies and found very similar results in cat IC. Winer et al. (1995) also found similar results with anti-GAD and anti-GABA antibodies in mustached bat auditory nuclei. The similarity of results with different antibodies suggests sufficient consistency for comparisons across studies and species.

Many of our observations included GAD-negative cells adjacent to (i.e., at the same depth in the tissue) as GAD+ cells. This suggests that the immunocytochemical reagents had access to the cells under observation and that the GAD-negative cell is non-GABAergic. As described in Methods, some of our sections had reduced or absent GAD staining in the deepest part of the tissue. We systematically excluded these regions from our analyses. We conclude that GAD+ cells are likely to be GABAergic. Beyond this, we conclude that most or all GAD-negative cells in the IC are glutamatergic. No IC neurons express glycine (Merchán et al., 2005) and few or none express acetylcholine, serotonin, dopamine, adrenaline, or noradrenaline (Klepper and Herbert, 1991; Tong et al., 2005; Motts et al., 2008). However, many IC cells express VGLUT2, identifying them as glutamatergic (Ito et al., 2011), and in a subsequent report Ito and Oliver (2012) considered all IC cells to be GABAergic or glutamatergic.

The tracer deposits were designed to be large in order to maximize the labeling of tectothalamic cells. This approach is important for both the assessment of GABAergic components of the pathway and for identification of collateral projections. The injections inevitably spread into adjacent nuclei in several cases. Most of these regions do not receive projections from the IC and so the tracer spread would be unlikely to have affected our results. Cases in which the deposit spread into the superior colliculus or nucleus of the brachium of the IC, which do receive projections from the IC, were excluded from the analysis. Our generation of similar results with multiple tracers strengthens our conclusions by reducing the possibility that a component of the pathway might be missed due to limitations of any single tracer. Indeed, we did observe quantitative differences across the tracers (different numbers of cells labeled) as is to be expected with these tracers (Schofield, 2008).

The use of anti-NeuN staining to identify neurons in the IC carries both advantages and disadvantages. NeuN is considered a neuron-specific marker that stains neuronal nuclei (Mullen et al., 1992). It frequently stains neuronal cytoplasm as well, though not as intensely as the nucleus. This stain was particularly helpful in estimating the percentage of IC neurons that are GABAergic because it helps to distinguish glial cells (NeuN-negative) from small neurons. As expected, all the GAD+ cells were also NeuN immunostained. This increased our confidence that our count of IC neurons did not include a substantial number of glial cells. The limitation is that there is no guarantee that the NeuN labels every neuron in the IC. In several brain locations, there are well known neurons that do not stain with NeuN (e.g., cerebellar Purkinje cells; ref). Our own observations suggest that the NeuN is not missing a major cell type in the IC, but we cannot rule out unintentional exclusion of a neuronal subtype.

### Functional implications and comparisons with earlier studies

We estimate that 21% of IC neurons are GABAergic in guinea pigs. This value is similar to results combined across the same 3 IC subdivisions in rats (~25%; Merchán et al., 2005). Data from other species are limited to the ICc, where 20% of ICc cells are GABAergic (cats: Oliver et al., 1994; mustached bats: Winer et al., 1995). In summary, the limited data that are available suggest relatively stable values across species for the percentage of GABAergic cells. Despite this consistency, the relative contributions of the GABAergic cells to the tectothalamic pathways may be more variable across species.

**Figure 6:**
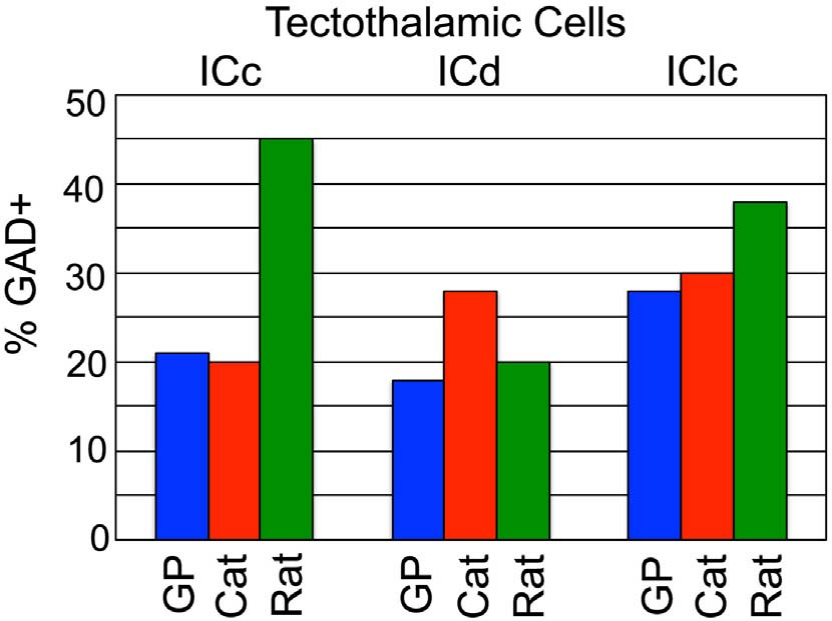
**Graph comparing the GABAergic tectothalamic cells across subdivisions of the IC between guinea pig, cat, and rat**. Data were obtained from current study (guinea pig), Winer et al., 1996 (cat), Peruzzi et al., 1997 (rat).

The current data indicate that ~22% of tectothalamic cells are GABAergic in guinea pigs (we restrict the discussion here to the ipsilateral pathway because there are no quantitative data on the GABAergic component of the contralateral pathway in other species). This value is similar to that in cats but rather different from rats (the two other species for which similar data are available; Figure 6). As mentioned in the Introduction, it was postulated that a high percentage of GABAergic tectothalamic cells may compensate for the relative absence of interneurons (Coomes et al., 2002). In cats, 25% of MG neurons are GABAergic interneurons (Huang et al., 1999), and about 20% of the tectothalamic cells are GABAergic. In contrast, rats have few GABAergic interneurons in the MG (<1% of MG neurons; Winer and Larue, 1988) but a higher proportion (~40%) of tectothalamic cells that are GABAergic (Peruzzi et al., 1997). The guinea pig MG also has very few GABAergic cells (unpublished observations) so we hypothesized that the percentage of tectothalamic cells that are GABAergic would be similar to what is reported in rat. However, we found that only 22% of the tectothalamic cells were GAD+, a value substantially lower than that in rats (Figure 6). Instead, our data suggest that guinea pig is quantitatively similar to the cat (Figure 6). Any broad conclusion will require data from more species, but the preliminary conclusion is that a high proportion of GABAergic cells in the tectothalamic pathway is not simply “compensating” for a lack of interneurons in the MG.

Interestingly, the IC subdivision with the highest percentage of GABAergic tectothalamic cells in the guinea pig was the IClc, with 28% of the cells GAD+. Once again, guinea pigs are more similar to cats (30%) than to rats (37%) (Figure 6). Examination of the ICd, however, prevents a simple analogy between species. Here, guinea pigs and rats are more similar, with cats being the outlier (Figure 6). In the end, the GABAergic component of the tectothalamic pathway does not appear to be directly related to the abundance of interneurons in the MG. What other factors or functions may relate to this variation remain to be identified.

Contralateral tectothalamic projections have been described in numerous species (see review: Wenstrup, 2005) but rarely receive much attention. We demonstrate that contralateral projections exist in guinea pigs and include both GABAergic and non-GABAergic cells. A similar projection appears in cats (Winer et al., 1996). The contralateral projection is significant in light of the common assertion that the IC is the last opportunity (below cortex) for bilateral interactions in the ascending auditory pathways. Certainly the convergence of ipsilateral and contralateral projections onto a single MG cell would provide opportunities for further “bilateral” interactions in the thalamus.

Additional speculation on the function of the GABAergic tectothalamic pathway has focused on the largest GABAergic tectothalamic cells. These large cells are presumably the source of the large GABAergic axons in the brachium of the IC (Saint Marie et al., 1997) and most likely underlie the short latency inhibition seen in the MG after stimulation of the brachium (Peruzzi et al., 1997). An abundance of VGLUT2 axosomatic endings on large GABAergic cells may help them to fire at short latency, allowing inhibition to reach the MG before the excitation from smaller, non-GABAergic tectothalamic cells (Ito et al., 2009). This suggestion is supported by recent experiments by Geis and Borst (2012), who recorded *in vivo* from large GABAergic cells in the IC dorsal cortex in mice. The function of this early inhibition is unclear, but may include a role in gating sound-evoked responses or controlling the latency of MG thalamocortical cells (Winer et al., 1996; Peruzzi et al., 1997; Bartlett and Smith, 1999; Geis and Borst, 2012; Venkataraman and Bartlett, 2013). Whether such functions are specifically related to the large GABAergic cells, or even whether the large and small cells have different functions, remains to be determined.

## Author contributions

Designed research, wrote the paper: Jeffrey G. Mellott, Brett R. Schofield; performed research, analyzed data: all authors.

### Conflict of interest statement

The authors declare that the research was conducted in the absence of any commercial or financial relationships that could be construed as a potential conflict of interest.

## Abbreviations

APT: anterior pretectum
Aq: cerebral aqueduct
DLL: dorsal nucleus of the lateral lemniscus
FB: Fast Blue
FG: FluoroGold
GAD: glutamic acid decarboxylase
IC: inferior colliculus
ICc: central nucleus of the inferior colliculus
ICd: dorsal cortex of the inferior colliculus
IClc: lateral cortex of the inferior colliculus
LG: lateral geniculate nucleus
LP: lateral posterior nucleus
MG: medial geniculate body
MGd: dorsal division of medial geniculate
MGm: medial division of medial geniculate
MGsg: suprageniculate division of medial geniculate
MGv: ventral division of medial geniculate
Neu-N: neuronal nuclei antibody
RB: Red Beads
s: shell of MG
SC: superior colliculus
scp: superior cerebellar peduncle
VLL: ventral nucleus of the lateral lemniscus.
